# Head-to-Head Comparison of Sedation and Somnolence Among 37 Antipsychotics in Schizophrenia, Bipolar Disorder, Major Depression, Autism Spectrum Disorders, Delirium, and Repurposed in COVID-19, Infectious Diseases, and Oncology From the FAERS, 2004–2020

**DOI:** 10.3389/fphar.2021.621691

**Published:** 2021-03-25

**Authors:** Andy R. Eugene, Beata Eugene, Marek Masiak, Jolanta Sylwia Masiak

**Affiliations:** ^1^ Independent Neurophysiology Unit, Department of Psychiatry, Medical University of Lublin, Lublin, Poland; ^2^ Medical Center, Lublin, Poland; ^3^ II Department of Psychiatry and Psychiatric Rehabilitation, Medical University of Lublin, Lublin, Poland

**Keywords:** sedation, sleep, pharmacogenomics, treatment resistant, major tranquilizers, COVID-19, SARS-CoV-2, psychiatry

## Abstract

**Objective: **Antipsychotic compounds are known to induce sedation somnolence and have expanded clinical indications beyond schizophrenia to regulatory approval in bipolar disorder, treatment-resistant depression, and is being repurposed in infectious diseases and oncology. However, the medical sciences literature lacks a comprehensive association between sedation and somnolence among a wide-range of antipsychotic compounds. The objective of this study is to assess the disproportionality of sedation and somnolence among thirty-seven typical and atypical antipsychotics.

**Materials and Methods: **Patient adverse drug reactions (ADR) cases were obtained from the United States Food and Drug Administration Adverse Events Reporting System (FAERS) between January 01, 2004 and September 30, 2020 for a wide-array of clinical indications and off-label use of antipsychotics. An assessment of disproportionality were based on cases of sedation and somnolence and calculated using the case/non-case methodology. Statistical analysis resulting in the reporting odds-ratio (ROR) with corresponding 95% confidence intervals (95% CI) were conducted using the R statistical programming language.

**Results: **Throughout the reporting period, there were a total of 9,373,236 cases with 99,251 specific ADRs reporting sedation and somnolence. Zuclopenthixol (*n* = 224) ROR = 13.3 (95% CI, 11.6–15.3) was most strongly associated of sedation and somnolence and haloperidol decanoate long-acting injection (LAI) was not statistically associated sedation and somnolence. Further, among atypical antipsychotic compounds, tiapride and asenapine were the top two compounds most strongly associated with sedation and somnolence. Comprehensively, the typical antipsychotics ROR = 5.05 (95%CI, 4.97–5.12) had a stronger association with sedation and somnolence when compared to atypical antipsychotics ROR = 4.65 (95%CI, 4.47–4.84).

**Conclusion: **We conducted a head-to-head comparison of thirty-seven antipsychotics and ranked the compounds based on the association of sedation and somnolence from ADR data collected throughout 16 years from the FAERS. The results are informative and with recent interests in repurposing phenothiazine antipsychotics in infectious disease and oncology provides an informative assessment of the compounds during repurposing and in psychopharmacology.

## Introduction

Antipsychotics were originally termed *major tranquilizers* as these compounds are effective in abating psychosis, mania, agitation, and aggression with also having an underlying pharmacodynamic outcome of inducing sedation and somnolence (Welsh, 1964). Major tranquilizers modulate monoaminergic targets including: adrenergic, noradrenergic, dopaminergic, histaminergic, and serotonergic receptors to induce sedation and somnolence (Welsh, 1964). Among the other terms used to describe antipsychotics, such as neuroleptics and ataraxic, the major tranquilizers were then re-classified to typical and atypical antipsychotics. Typical antipsychotics are ligands which predominately bind to dopamine D2-receptors, whereas, atypical antipsychotics are ligands with higher binding affinities towards the various subtypes of serotonin 5-HT receptors and may be dichotomized using the ratio of 5-HT2 to dopamine D2 binding affinities (Meltzer et al., 1989). Despite the re-classification of antipsychotics, one common theme is the clinical outcome of producing sedation and somnolence.

In psychiatry, sleep dysregulation is common in patients experiencing psychosis, mania, depression, delirium, suicidal ideation, irritability, delusions, agitation, and other disruptions in mental health. Sedation and somnolence leading to proper sleep is neuroprotective and with increasing interest in repurposing antipsychotics in oncology and infectious diseases meets an unmet clinical need in the medical sciences and patient care (Koyanagi and Stickley, 2015; Laskemoen et al., 2019). Research has shown that with sufficient sleep, one experiences enhanced cognition coupled with efficient memory recall leading to improved academic performance; while in contrast, when sleep is chronically deficient, one may experience impaired memory, depression, anxiety, persecutory ideation, cognitive disorganization, paranoia, and hallucinations (Kelly et al., 2001; Alhola and Polo-Kantola, 2007; Freeman et al., 2010; Reeve et al., 2018; Eugene, 2019; Eugene, 2020a).

Antipsychotic compounds have expanded regulatory approval beyond schizophrenia as demonstrated by clinical studies showing benefit in augmenting antidepressants in treatment-resistant depression, as well as is indicated in patients with bipolar mania, bipolar depression, and mixed features in bipolar disorder (Eugene and Masiak, 2017; Eli Lilly and Company, 2019; AstraZeneca Pharmaceuticals, 2020; America Pharmaceutical, 2020a; Otsuka America Pharmaceutical, 2020a; Otsuka America Pharmaceutical, 2020b). However, despite the wide-spread use of major tranquilizers, there is a fundamental gap in psychopharmacology with ranking a substantive number of antipsychotics based on association of sedation and somnolence.

The most comprehensive study, to date, specifically assessing somnolence in antipsychotics was conducted by Fang and colleagues and includes 12 compounds (Fang et al., 2016). In that study, which evaluated the absolute risk increase of somnolence showed that clozapine being classified as high somnolence, while olanzapine, perphenazine, quetiapine, risperidone, and ziprasidone classified as moderate somnolence, and lastly, asenapine, aripiprazole, cariprazine, lurasiodone, haloperidol, and paliperidone were categorized as low somnolence compounds (Fang et al., 2016). In a post-hoc analysis by Gao and colleagues evaluating the incidence of somnolence in ten clinical trials (*n* = 4,786) of four total antipsychotics, reported that out of asenapine, olanzapine, risperidone, and haloperidol, only olanzapine and asenapine showed significantly increased rates of somnolence relative to placebo (Gao et al., 2013). The most comprehensive study evaluating sedation, to date, is a network meta-analysis by Huhn and colleagues, which showed out of thirty-two antipsychotics, zuclopenthixol had the highest risk of sedation (Huhn et al., 2019). Taken together, these findings suggest that the wide inter-individual variability in outcomes of sedation and somnolence and may be due to drug-gene influences, drug-drug interactions (DDI), or combination of both influencing drug pharmacokinetics – via DNA sequence variants influencing cytochrome P450 (CYP) enzymes – and pharmacodynamics.

Studies using electroencephalography brain-mapping demonstrate that antipsychotics are associated with the delta frequency band and slow wave sleep via the serotonin 5-HT2A and 5-HT2C receptors (Sharpley et al., 1994; Eugene et al., 2014; Eugene and Masiak, 2014). Whereas, other translational biomarker studies, using prepulse inhibition, reports that sleep deprivation induces perceptual distortion (e.g. auditory hallucinations), cognitive disorganization, and anhedonia among healthy adults (Petrovsky et al., 2014). With this information as a background, the objective of this study is to assess the disproportionality in reporting of sedation and somnolence among thirty-seven typical and atypical antipsychotic drugs using post-marketing adverse drug events reported to the United States Food and Drug Administration.

## Materials and Methods

Patient adverse drug reaction (ADR) cases were obtained from the United States Food and Drug Administration Adverse Events Reporting System (FAERS) and assessed retrospectively in an observational study approach. The first recorded case using the term ‘somnolence’, in the FAERS, was in 1993 and the first record of ‘sedation’ in the FAERS was in 1969. This study includes all cases of either sedation or somnolence from the January 01, 2004 to the September 30, 2020 contained no identifiable patient information in the FAERS reports. The Medical Dictionary for Regulatory Activities (MedDRA) coded preferred terms used in this study are either sedation or somnolence and no other ADR was considered for analysis (Brown et al., 1999). For the thirty-seven antipsychotic compounds included in this study, the primary outcome variable is the reporting odds-ratio and this method has been previously reported in studies from the first author (Eugene and Eugene, 2018; Eugene, 2019; Eugene, 2020a).

### List of Documented Indications Reported to the FAERS

A complete list of clinical indications corresponding to the study data presented may be seen in the supplementary files. A partial list is as follows: schizophrenia, bipolar disorder, depression, anxiety, hallucination, sleep disorder, attention deficit/hyperactivity disorder, agitation, aggression, parkinson's disease psychosis, insomnia, mania, suicide attempt, paranoia, autism spectrum disorder, dementia, oppositional defiant disorder, delirium, post-traumatic stress disorder, tourette's disorder, adjuvant therapy, generalised anxiety disorder, impulsive behavior, sedation, psychomotor hyperactivity, intellectual disability, asperger's disorder, sleep disorder therapy, self injurious behavior, substance-induced psychotic disorder, vomiting, sedative therapy, off label use, prophylaxis of nausea and vomiting, huntington's disease, hyperkinesia, developmental delay, postpartum depression, renal cancer, antisocial behaviour, creutzfeldt-jakob disease, tic tourette's disorder, and traumatic brain injury.

### List of Thirty-Seven Antipsychotic Compounds

The following antipsychotic compound generic names were included in the study: amisulpride, aripiprazole, aripiprazole lauroxil, asenapine, blonanserin, brexpiprazole, cariprazine, chlorpromazine, chlorprothixene, clozapine, cyamemazine, droperidol, fluphenazine, haloperidol, haloperidol decanoate, iloperidone, loxapine, lurasidone, melperone, olanzapine, paliperidone, paliperidone palmitate, periciazine, perphenazine, pimavanserin, pimozide, pipamperone, prochlorperazine, promazine, quetiapine, risperidone, thioridazine, thiothixene, tiapride, trifluoperazine, ziprasidone, and zuclopenthixol.

### Statistical Analysis

Disproportionality signal analysis of antipsychotic cases reporting somnolence was calculated using the reporting odds-ratio (ROR) with the corresponding 95% confidence interval (CI). The ROR is the odds of a certain event occurring for a medicinal product, compared to the odds of the same event occurring with all other medicinal products in the database. This case/non-case method, similar to a case-control study, via a two-by-two contingency table is calculated by the *fisher.test* command in R. For study inclusion, a minimum of one adverse event case was required. All computations and the figure illustration were performed using R (version 4.0.2, R Foundation for Statistical Computing, Vienna, Austria) (Team, 2013). A signal is considered statistically significant when the lower limit of the 95% CI of the ROR is greater than one.

## Results

From January 2004 to September 30, 2020, there were a total of 9,373,236 overall cases reported to the FAERS and 99,251 cases specifically were reported as sedation and somnolence. Among the thirty-seven antipsychotic compounds, quetiapine (*n* = 96,985), an atypical antipsychotic, has the highest number of reported cases; whereas, haloperidol decanoate (*n* = 6), a typical antipsychotic in a long-acting injection formulation, had the least number of cases. The top ranked antipsychotics with the strongest association of sedation and somnolence were: zuclopenthixol (*n* = 224) ROR = 13.3 (95% CI, 11.6–15.3), tiapride (*n* = 76) ROR = 11.8 (95% CI, 9.3–15.0), and cyamemazine (*n* = 245) ROR = 10.7 (95% CI, 9.4–12.2). The long-acting injection formulation, haloperidol decanoate (*n* = 6) ROR = 1.7 (95% CI, 0.8–3.8) was not associated with sedation or somnolence; while, in contrast the pill version of haloperidol (*n* = 1158) had a strong association with a ROR = 5.6 (95% CI, 5.3–6.0).

The following three antipsychotic compounds were least associated with sedation and somnolence (ROR crosses 2): prochlorperazine (*n* = 202) ROR = 1.4 (95% CI, 1.2–1.6), paliperidone (*n* = 641) ROR = 1.9 (95% CI, 1.8–2.0), and aripiprazole lauroxil (*n* = 36) ROR = 2.1 (95% CI, 1.5–3.0). Of note, as with the pill formulation of haloperidol, the aripiprazole pill formulation had a stronger association with sedation and somnolence (*n* = 2,647) ROR = 3.7 (95% CI, 3.6–3.9), when compared to the long-acting injection formulations of the two compounds. Overall, the typical (ROR = 5.05 [95% CI, 4.97–5.12]) antipsychotics had a stronger association with sedation and somnolence when compared to atypical (ROR = 4.65 [95% CI, 4.47–4.84]) antipsychotics.

**FIGURE 1:**
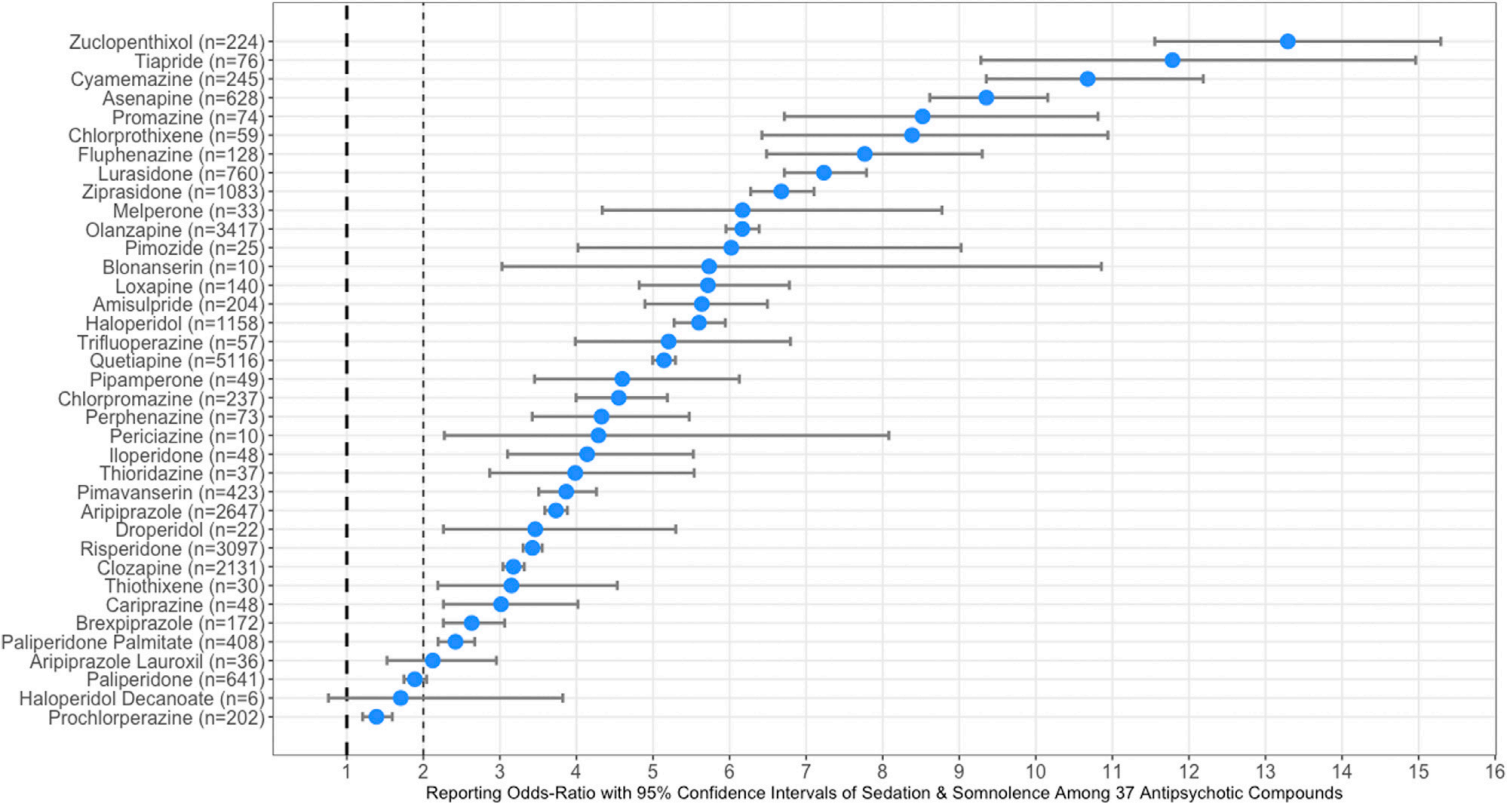
Forest plots illustrating reporting odds-ratios (RORs) with 95% confidence intervals (CI) of sedation and somnolence among all thirty-seven typical an atypical antipsychotic compounds. The dashed line at one represents the reference point for statistical significance of sedation and somnolence.

Figure 1 illustrates the forest plots illustrating RORs with corresponding 95% CIs for antipsychotic compounds, sorted from the highest to lowest of sedation and somnolence. In Figure 1, the dashed line at 1 represents the reference point for statistical significance for sedation and somnolence. A list of the thirty-seven antipsychotic compounds sorted from highest to lowest reporting odds of sedation and somnolence are provided as a Supplementary File S2.

## Discussion

### Study Findings and Recent Interests in Antipsychotic Drug Repurposing

In this pharmacovigilance study with adverse drug reactions spanning sixteen-years of reports to the United States Food and Drug Administration, thirty-five of the thirty-seven typical and atypical antipsychotic formulations showed statistical significance with sedation and somnolence. As mentioned, the typical antipsychotics had a stronger association with sedation and somnolence as compared to the atypical antipsychotics. We found zuclopenthixol ranked highest in association with sedation and somnolence and our findings are consistent with a large-scale network meta-analysis which reported zuclopenthixol also being ranked first among 32 antipsychotic drugs (Huhn et al., 2019). The methodology used in this study for a direct head-to-head comparison of drugs reported to the FAERS provides a pragmatic approach in clinical pharmacology to assess signal strength of ADRs beyond traditional studies using meta-analyses.

Compound metabolites are of increasing importance to clinical pharmacologists due to an increasing interests in pharmacogenomics and drug-interactions associated CYP enzymes, as well as polymorphisms of receptors, transporters, and also transcription factors influencing CYP enzymes (Qin et al., 2019). Risperidone is metabolized via CYP2D6 to paliperidone and both compounds are represented here as independent compounds with the paliperidone pill formulation having a weaker association with sedation and somnolence when compared to the parent compound risperidone. Another compound with a well-known metabolite is loxapine, and a previous study showed amoxapine, a metabolite of loxapine, ranked number one among thirty antidepressants in reporting odds of somnolence (Eugene, 2020a). The current study results are informative, especially in patients who experience acute agitation and aggression associated with mania and psychosis. For example, the most recent approved inhaled formulation of loxapine has a median time to the maximum plasma concentration (Tmax) of 2 min and reported to be effective in acute agitation in bipolar I disorder and schizophrenia (Galen, 2019). The capsule formulation of loxapine is reported to cause sedation within 20 to 30 min (Actavis Pharma, 2016).

Zuclopenthixol, the neuroleptic with the strongest association with sedation and somnolence, has emerged as a compound that shows *in vitro* inhibition of the severe acute respiratory coronavirus 2 (SARS-CoV-2) pathogen which causes the novel coronavirus disease 2019 (COVID-19) (Bocci et al., 2020). A recent review article reported thioridazine, fluphenazine, perphenazine, chlorpromazine, prochlorperazine inhibits RNA viruses as these phenothiazines inhibit cell-cell fusion, viral replication, clathrin-dependent endocytosis, and entry into cells (Otręba et al., 2020). Dyall and colleagues, reported triflupromazine inhibits the middle east respiratory syndrome coronavirus (MERS-CoV) and the SARS-CoV-1 (Dyall et al., 2014). Another study, found trifluoperazine inhibits lung metastasis, bone metastasis, and brain metastasis of melanoma via the mechanism of cell cycle arrest at the G0/G1 phase and mitochondrial-dependent intrinsic apoptosis (Xia et al., 2020). Via the same mechanism, another study described fluphenazine suppresses growth of triple negative breast cancer, as well as lung and brain metastasis in a subcutaneous xenograft model (Xu et al., 2019). Another phenothiazine, perphenazine, was shown to inhibit growth of progesterone-receptor resistant endometrial cancer (Chen et al., 2020). The anticancer properties of antipsychotics, more particularly, the typical antipsychotics are described being related to the dopamine D2-receptor antagonist properties which disrupt critical metabolic processes in tumors and cancer cells (Weissenrieder et al., 2019).

Researchers in France reported that chlorpromazine, a derivative of methylene blue, inhibits SARS-CoV-2 *in vitro* and other studies have shown clinical benefit of chlorpromazine in the most invasive glial tumor, the grade IV astrocytoma, glioblastoma multiforme (Amaral et al., 2004; Kamarudin and Parhar, 2019; Abbruzzese et al., 2020; Plaze et al., 2020). Methylene blue, the first synthetic compound in medicine has antimalarial properties, anti-SARS-Cov-2 properties, and a recent study reported the compound to be neuroprotective (López-Muñoz et al., 2005; Poteet et al., 2012; Gendrot et al., 2020). Other drug repurposing efforts identified haloperidol, a typical antipsychotic that is a ligand of the sigma-1 and sigma-2 receptors, also inhibits SARS-Cov-2 *in vitro* (Gordon et al., 2020). With the aforementioned efforts in repurposing neuroleptics in oncology and infectious disease, the results of this study will be informative to clinical research teams.

### Drug Interactions With Antidepressant Augmentation Using Antipsychotics

Antipsychotic drugs are increasingly being used to augment antidepressants. A classic example is the olanzapine/fluoxetine combination approved for treatment-resistant depression and bipolar depression (Eli Lilly and Company, 2018). Fluoxetine is strong inhibitor of CYP2C19 and CYP2D6 and will increase the area-under-the-concentration-time curve (AUC) of sensitive CYP2C19 or CYP2D6 substrates by greater than 5-fold drug exposure (United States Food and Drug Administration, 2020). Moreover, fluoxetine’s enantiomer metabolites (*R*)-norfluoxetine and (*S*)-norfluoxetine produce time-dependent inhibition of CYP3A4 (Ki = 8 µM) and CYP2C19 (Ki = 7 µM), respectively and has shown to inhibit SARS-Cov-2 (Lutz et al., 2013; Eugene, 2020b). Olanzapine is a substrate of CYP1A2 and should not be administered with strong CYP1A2 inhibitors to avoid excessive increases in AUC and Cmax. For example, fluvoxamine is a strong CYP1A2 and CYP2C19 inhibitor and would result in a drug-interaction increasing the AUC of asenapine, chlorpromazine, clozapine, olanzapine, and thiothixene. Among antipsychotics: aripiprazole, brexpiprazole, and quetiapine are also used in augmenting antidepressants. Both aripiprazole and brexpiprazole are CYP2D6 substrates and would result in a drug-drug-interaction (DDI) if combined with fluoxetine, paroxetine, sertraline, bupoprion, and duloxetine due to the antidepressants being CYP2D6 inhibitors. The pharmacokinetic outcomes of increased an AUC and Cmax would be even further exacerbated if the patient is a CYP2D6 Intermediate Metabolizer or CYP2D6 Poor Metabolizer (Hefner, 2018; Eugene, 2019).

Aside from being indicated in patients diagnosed with schizophrenia, quetiapine is indicated in patients diagnosed with manic episodes bipolar I disorder, as well as monotherapy in bipolar depression (AstraZeneca Pharmaceuticals, 2020). Further, quetiapine is biotransformed by CYP3A4 to norquetiapine, which has moderate binding affinity to the norepinephrine transporter (Ki = 12 nM) as is found in some traditional antidepressant compounds (Besnard et al., 2012). It is of benefit to recognize that strong CYP3A4 inhibitors include: boceprevir, cobicistat, clarithromycin, danoprevir and ritonavir, elvitegravir and ritonavir, grapefruit juice, indinavir and ritonavir, itraconazole, ketoconazole, lopinavir and ritonavir, paritaprevir and ritonavir and (ombitasvir and/or dasabuvir), posaconazole, ritonavir, saquinavir and ritonavir, telaprevir, tipranavir and ritonavir, telithromycin, troleandomycin, voriconazole, idelalisib, nefazodone, and nelfinavir (United States Food and Drug Administration, 2020). Moreover, a list of known strong and moderate CYP3A4 inducers are: apalutamide, carbamazepine, enzalutamide, mitotane, phenytoin, rifampin, St. John’s Wort, bosentan, efavirenz, etravirine, phenobarbital, and primidone (United States Food and Drug Administration, 2020).

### Study Limitations

The limitations of this observational study are that the cases are based on voluntary reporting of adverse drug events and are likely under-reported, resulting in the total number of cases not complete in patients treated with the tested compounds. Further, the results are indicative of pharmacovigilance signal strength and not causation. Our work does not factor in a potential dose-related propensity to induce sedation or somnolence as well as, does not factor in age, gender, or body-mass index. Nevertheless, the results are well catalogued, maintained, and available for public access within the U.S. FDA Adverse Events Reporting System. This also means that not all marketed antipsychotic compounds are included in this study, but the thirty-seven antipsychotics that are included provide an informative reference in clinical pharmacology, research, and medical education. Overall, the FAERS patient cases are a broad representative sample of the population and is reported by clinicians, pharmaceutical companies, and consumers.

## Conclusion

The main study findings, from this population-wide head-to-head comparison of thirty-seven antipsychotics, is that zuclopenthixol showed the strongest association with sedation and somnolence while prochloperazine resulted in the weakest association. Taken together, this study reports adverse drug reaction data collected throughout 16 years from the FDA Adverse Events Reporting System serving as a clinically relevant reference in psychiatry and in efforts of drug repurposing of antipsychotic compounds in other fields of medicine.

## Data Availability

Publicly available datasets were analyzed in this study. This data can be found here: https://fis.fda.gov/extensions/FPD-QDE-FAERS/FPD-QDE-FAERS.html.
