# Supplementary material for: Head-to-Head Comparison of Sedation and Somnolence Among 37 Antipsychotics in Schizophrenia, Bipolar Disorder, Major Depression, Autism Spectrum Disorders, Delirium, and Repurposed in COVID-19, Infectious Diseases, and Oncology From the FAERS, 2004–2020
Source: Front Pharmacol. 2021 Mar 25;12:621691. doi: 10.3389/fphar.2021.621691 (PMC8027114; doi:10.3389/fphar.2021.621691)
Supplement: Supplementary file 1 [file datasheet1.docx]

**Supplementary File**

**Indications for Antipsychotic Compounds Reported to the FAERS from 2004 to 2020**

schizophrenia

product used for unknown indication

bipolar disorder

psychotic disorder

depression

schizoaffective disorder

anxiety

hallucination

drug use for unknown indication

sleep disorder

bipolar i disorder

delusion

attention deficit/hyperactivity disorder

agitation

aggression

parkinson's disease psychosis

major depression

affective disorder

mental disorder

insomnia

mania

abnormal behaviour

parkinson's disease

suicide attempt

paranoia

autism spectrum disorder

obsessive-compulsive disorder

intentional overdose

dementia

oppositional defiant disorder

delirium

bipolar ii disorder

seizure

post-traumatic stress disorder

poisoning deliberate

nausea

tourette's disorder

schizoaffective disorder bipolar type

drug abuse

anger

toxicity to various agents

mood swings

dementia alzheimer's type

ill-defined disorder

irritability

psychotic symptom

acute psychosis

restlessness

autism

panic attack

adjuvant therapy

borderline personality disorder

intentional self-injury

personality disorder

delusional disorder

accidental exposure

schizoaffective disorder depressive type

seasonal affective disorder

suicidal ideation

drug therapy

tic

generalised anxiety disorder

impulsive behaviour

stress

behaviour disorder

medication error

sedation

psychomotor hyperactivity

emotional disorder

screaming

tachyphrenia

cognitive disorder

encephalitis autoimmune

pain

schizophreniform disorder

intellectual disability

overdose

antidepressant therapy

anxiety disorder

asperger's disorder

disturbance in social behaviour

hypomania

sleep disorder therapy

dementia with lewy bodies

panic disorder

accidental exposure to product

intermittent explosive disorder

confusional state

mood altered

convulsion

product administration error

thinking abnormal

chorea

learning disorder

migraine

mixed anxiety and depressive disorder

schizoaffective disorder schizophrenia

self injurious behaviour

substance-induced psychotic disorder

accidental exposure to product by child

conduct disorder

drug administration error

histrionic personality disorder

impulse-control disorder

vomiting

epilepsy

prophylaxis

psychotherapy

behavioural and psychiatric symptoms of dementia

emotional distress

nervous system disorder

adjustment disorder

craniocerebral injury

depressive symptom

foetal exposure during pregnancy

gout

insomnia irritability

intentional product use issue

patient restraint

postoperative delirium

psychotic disorder due to a general medical condition

senile dementia

somnolence

apathy

compulsions

depressed mood

hiccups

intrusive thoughts

neuropsychiatric symptoms

relaxation therapy

somatic delusion

tardive dyskinesia

bipolar disorder schizoaffective disorder

catatonia

drug dispensing error

drug prescribing error

hypersomnia

initial insomnia

maternal exposure during breast feeding

psychiatric symptom

sedative therapy

vascular dementia

affect lability

agitated depression

bipolar disorder mania

bipolar disorder psychotic disorder

disturbance in attention

drug dependence

drug exposure during pregnancy

fibromyalgia

malaise

merycism

negative thoughts

nervousness

nightmare

off label use

persecutory delusion

prophylaxis of nausea and vomiting

schizotypal personality disorder

sexual inhibition

substance abuse

tremor

wrong patient received product

wrong product administered

adverse event

agitation dementia

anorexia nervosa

appetite disorder

attention deficit/hyperactivity disorder bipolar disorder

cyclothymic disorder

flight of ideas

hepato-lenticular degeneration

huntington's disease

hyperkinesia

multiple sclerosis

narcolepsy

psychiatric disorder prophylaxis

senile psychosis

substance dependence

wrong patient received medication

agitation anxiety

amnesia

back pain

ballismus

bipolar disorder insomnia

conversion disorder

decreased appetite

dissociative disorder

dissociative identity disorder

drug use disorder

dyskinesia

eating disorder

hashimoto's encephalopathy

molluscum contagiosum

mood disorder due to a general medical condition

obsessive thoughts

polydipsia psychogenic

psychiatric decompensation

psychotic behaviour

schizoid personality disorder

sleep apnoea syndrome

speech disorder

stereotypy

wrong drug administered

accidental overdose

agitation mania

alcohol abuse

analgesic therapy

anorexia

anxiety bipolar disorder depression

anxiety bipolar disorder schizophrenia tourette's disorder

anxiety depression

bipolar disorder borderline personality disorder post-traumatic stress disorder

bipolar disorder schizophrenia

bipolar i disorder schizoaffective disorder

borderline mental impairment

brief psychotic disorder

dementia psychotic disorder

depersonalisation

depression obsessive-compulsive disorder

developmental delay

disorientation

drug withdrawal syndrome

dystonia

encephalopathy

euphoric mood

extrapyramidal disorder

factitious disorder

fear

feeling abnormal

headache

incorrect dose administered

intentional product misuse

irritable bowel syndrome

lethargy

logorrhoea

mania psychotic disorder

mental disorder due to a general medical condition

mental retardation

multiple drug overdose

narcissistic personality disorder

obsessive-compulsive personality disorder

paedophilia

parkinsonism

partner stress

poisoning

postpartum depression

postpartum disorder

premedication

product use in unapproved indication

psychotic disorder schizoaffective disorder

rebound psychosis

renal cancer

sleep terror

sluggishness

social avoidant behaviour

social phobia

substance-induced mood disorder

suicidal behaviour

unevaluable therapy

abdominal discomfort

abnormal behaviour aggression agitation

abnormal dreams

accidental drug intake by child

adhd mood disorder nos

adjustment disorder with mixed anxiety and depressed mood

affective disorder anxiety

affective disorder attention deficit/hyperactivity disorder

aggression agitation

aggression bipolar disorder

aggression paranoia

aggression psychotic disorder

agitation delirium

agitation postoperative

agitation sedation

agoraphobia

anger anxiety

angiopathy

antiemetic supportive care

antisocial behaviour

anxiety attention deficit/hyperactivity disorder obsessive-compulsive disorder

anxiety bipolar disorder tachyphrenia

anxiety bipolar i disorder schizoaffective disorder

anxiety cognitive disorder depression

anxiety delusion depression

anxiety hallucinations

anxiety insomnia

anxiety intentional self-injury

anxiety off label use

anxiety sleep disorder

attention deficit/hyperactivity disorder depression

attention deficit/hyperactivity disorder mood swings

auditory disorder

behavioural therapy

bipolar affective disorder

bipolar disorder generalised anxiety disorder

bipolar disorder hallucination

bipolar disorder irritability

bipolar disorder major depression

bipolar disorder major depression psychotic behaviour

bipolar disorder mania psychotic disorder

bipolar disorder pulmonary embolism

bipolar disorder sleep disorder

bipolar disorder thyroid function test abnormal

bipolar i disorder insomnia

bipolar i disorder mania

bipolar i disorder psychotic disorder

blood cholesterol

blood pressure abnormal

blood pressure decreased

brain injury

cerebrovascular accident

completed suicide

complex regional pain syndrome

creutzfeldt-jakob disease

crying

cyclic vomiting syndrome

delirium delusion

delirium dementia

delirium dementia alzheimer's type

delusion dementia

delusion hallucination parkinson's disease

delusion of replacement

delusion schizophrenia

dementia alzheimer's type psychotic disorder

dementia paranoia

dementia psychiatric symptom

dementia with lewy bodies hallucination parkinson's disease

dependence

depression mental disorder schizophrenia

depression post-traumatic stress disorder

depression suicidal

depressive delusion

depressive disorder

disability

disinhibition

disruptive mood dysregulation disorder

dizziness

drug therapy enhancement

drug therapy somnolence

dysarthria

dysphemia

dysphoria

dysthymic disorder

emotional distress surgery

emotional poverty

endoscopic retrograde cholangiopancreatography

exposure via breast milk

eye laser surgery

family stress

fatigue

feeling of relaxation

frontotemporal dementia

frustration

generalized anxiety disorder

grand mal convulsion

grandiosity

hallucinations

homicidal ideation

hostility

hot flush

hyperprolactinaemia

hypertension

hypervigilance

hypnagogic hallucination

infection

infection schizoaffective disorder

injury

insomnia related to another mental condition

kabuki make-up syndrome

labyrinthitis

learning disability

loss of consciousness

major depression off label use

major depression psychotic disorder

mania schizoaffective disorder

memory impairment

mental status changes

metal poisoning

middle insomnia

mild mental retardation

mood altered post-traumatic stress disorder

movement disorder

muscle spasms

neuralgia

nightmare post-traumatic stress disorder sleep disorder

obsessive-compulsive disorder tic

oropharyngeal pain

paranoia schizophrenia

persistent depressive disorder

personality change due to a general medical condition

premenstrual syndrome

pressure of speech

prophylactic chemotherapy

prophylaxis against transplant rejection

prophylaxis psychotic disorder

psychological abuse

psychotic disorder schizophrenia

psychotic disorder thinking abnormal

rapid eye movements sleep abnormal

refusal of treatment by patient

sexual abuse

sexually inappropriate behaviour

social anxiety disorder

somatic symptom disorder

substance use

suspiciousness

tension

tension headache

therapy regimen changed

tic tourette's disorder

tooth abscess

transient psychosis

traumatic brain injury

unevaluable event

vestibular neuronitis

victim of sexual abuse

violence-related symptom

vomiting in pregnancy

withdrawal syndrome
