# Supplementary material for: Head-to-Head Comparison of Sedation and Somnolence Among 37 Antipsychotics in Schizophrenia, Bipolar Disorder, Major Depression, Autism Spectrum Disorders, Delirium, and Repurposed in COVID-19, Infectious Diseases, and Oncology From the FAERS, 2004–2020
Source: Front Pharmacol. 2021 Mar 25;12:621691. doi: 10.3389/fphar.2021.621691 (PMC8027114; doi:10.3389/fphar.2021.621691)
Supplement: Supplementary file 2 [file table1.docx]

| Supplementary Table 1: Reporting Odds-Ratios (ROR) and 95% confidence intervals (CI) of 37 typical (n=18) and atypical (n=19) antipsychotic compounds. | | | | | |
| --- | --- | --- | --- | --- | --- |
| Antipsychotic Compound | **Sedation & Somnolence Cases** | **Other Cases** | **ROR** | **95% CI** | **Antipsychotic Classification** |
| Zuclopenthixol | 224 | 1578 | 13.3 | 11.6-15.3 | Typical |
| Tiapride | 76 | 603 | 11.8 | 9.3-15.0 | Atypical |
| Cyamemazine | 245 | 2149 | 10.7 | 9.4-12.2 | Typical |
| Asenapine | 628 | 6309 | 9.4 | 8.6-10.2 | Atypical |
| Promazine | 74 | 812 | 8.5 | 6.7-10.8 | Typical |
| Chlorprothixene | 59 | 658 | 8.4 | 6.4-10.9 | Typical |
| Fluphenazine | 128 | 1542 | 7.8 | 6.5-9.3 | Typical |
| Lurasidone | 760 | 9884 | 7.2 | 6.7-7.8 | Atypical |
| Ziprasidone | 1083 | 15300 | 6.7 | 6.3-7.1 | Atypical |
| Melperone | 33 | 500 | 6.2 | 4.3-8.8 | Atypical |
| Olanzapine | 3417 | 53322 | 6.2 | 6.0-6.4 | Atypical |
| Pimozide | 25 | 388 | 6 | 4.0-9.0 | Typical |
| Blonanserin | 10 | 163 | 5.7 | 3.0-10.9 | Atypical |
| Loxapine | 140 | 2291 | 5.7 | 4.8-6.8 | Typical |
| Amisulpride | 204 | 3387 | 5.6 | 4.9-6.5 | Atypical |
| Haloperidol | 1158 | 19509 | 5.6 | 5.3-6.0 | Typical |
| Trifluoperazine | 57 | 1024 | 5.2 | 4.0-6.8 | Typical |
| Quetiapine | 5116 | 96985 | 5.1 | 5.0-5.3 | Atypical |
| Pipamperone | 49 | 996 | 4.6 | 3.5-6.1 | Typical |
| Chlorpromazine | 237 | 4874 | 4.6 | 4.0-5.2 | Typical |
| Perphenazine | 73 | 1577 | 4.3 | 3.4-5.5 | Typical |
| Periciazine | 10 | 218 | 4.3 | 2.3-8.1 | Typical |
| Iloperidone | 48 | 1084 | 4.1 | 3.1-5.5 | Atypical |
| Thioridazine | 37 | 868 | 4 | 2.9-5.5 | Typical |
| Pimavanserin | 423 | 10257 | 3.9 | 3.5-4.3 | Atypical |
| Aripiprazole | 2647 | 67604 | 3.7 | 3.6-3.9 | Atypical |
| Droperidol | 22 | 594 | 3.5 | 2.3-5.3 | Typical |
| Risperidone | 3097 | 86400 | 3.4 | 3.3-3.6 | Atypical |
| Clozapine | 2131 | 63631 | 3.2 | 3.0-3.3 | Atypical |
| Thiothixene | 30 | 890 | 3.2 | 2.3-4.5 | Typical |
| Cariprazine | 48 | 1488 | 3 | 2.3-4.0 | Atypical |
| Brexpiprazole | 172 | 6115 | 2.6 | 2.3-3.1 | Atypical |
| Paliperidone Palmitate | 408 | 15789 | 2.4 | 2.2-2.7 | Atypical |
| Aripiprazole Lauroxil | 36 | 1585 | 2.1 | 1.5-3.0 | Atypical |
| Paliperidone | 641 | 31819 | 1.9 | 1.8-2.0 | Atypical |
| Haloperidol Decanoate | 6 | 329 | 1.7 | 0.8-3.8 | Typical |
| Prochlorperazine | 202 | 13619 | 1.4 | 1.2-1.6 | Typical |
